# Retraction: Exploration of inhibitory mechanisms of curcumin in lung cancer metastasis using a miRNA- transcription factor-target gene network

**DOI:** 10.1371/journal.pone.0189070

**Published:** 2017-11-30

**Authors:** De-min Jiao, Li Yan, Li-shan Wang, Hui-zhen Hu, Xia-li Tang, Jun Chen, Jian Wang, You Li, Qing-yong Chen, PLOS ONE Editors

After publication of this article, the corresponding author, Qing-yong Chen, notified the editorial office that the curcumin used in this study was expired at the time of use. The authors expressed concern that this may have affected the results of the MTT assays and microarray expression experiments reported in the article; they are unable to repeat the experiments at this time. Due to concerns about the validity of the data and results reported in this work, the authors and the PLOS ONE Editors retract this article.
